# The Challenges and Knowledge Gaps in Malaria Therapy: A Stakeholder Approach to Improving Oral Quinine Use in the Treatment of Childhood Malaria in Ghana

**DOI:** 10.1155/2018/1784645

**Published:** 2018-11-14

**Authors:** Bartholomew Yir-Erong, Marcel Tunkumgnen Bayor, Isaac Ayensu, Stephen Yao Gbedema, Joshua Boateng

**Affiliations:** ^1^Department of Pharmaceutics, Kwame Nkrumah University of Science & Technology, Kumasi, Ghana; ^2^Department of Pharmaceutical Chemistry, Kwame Nkrumah University of Science & Technology, Kumasi, Ghana; ^3^Department of Pharmaceutical, Chemical and Environmental Sciences, University of Greenwich, Medway Campus, Kent ME4 4TB, UK

## Abstract

**Background:**

The study was undertaken to elicit the knowledge, views, and perceptions of key stakeholders on malaria, its bioburden, and treatment options, in order to ascertain the knowledge gabs and challenges, especially in the use of oral quinine in childhood malaria.

**Methods:**

A cross-sectional survey was conducted using a well-structured Likert Scale and self-administered questionnaire. The principal site of the study was a government-run children's hospital located in the Ashiedu Keteke Sub-Metro of Accra. The study population included health workers, parents, and guardians or care givers. The participants were 300, purposively selected, and consisted of both men (41%) and women (59%) who were twenty years and above, whether employed (42%), self-employed (37%), or unemployed (21%).

**Results:**

Majority of the participants (78%) demonstrated above average knowledge of malaria. However, their awareness of the causes, modes of transmission, signs, and symptoms as well as preventive mechanisms of malaria did not result in low incidence of malaria. About 77% of the respondents agreed they would seek treatment within 24 hours once signs and symptoms are detected. Though close to 50% undertook home treatment of malaria, majority eventually sought treatment at hospital or clinic. Above 92% of respondents knew that quinine is used to treat malaria and agreed its bitter taste greatly affects compliance, especially in children. Consequently, 95% of the respondents would be glad if its bitter taste is masked.

**Conclusion:**

The study demonstrated the availability of substantial knowledge of the devastating effects of malaria, especially in children. Therefore, there is the need to ensure the availability and utilization of effective paediatric formulations in the fight against malaria. From this study, fast dissolving oral thin film with a good mouth feel, would be the formulation of choice for quinine.

## 1. Introduction

Malaria is a serious and potentially fatal disease caused by the parasite,* Plasmodium*. There are four species of this parasite which cause the disease in humans, namely,* P*.* falciparum*,* P*.* vivax*,* P*.* malariae*, and* P*.* ovale. *These parasites are transmitted basically through the bites of female Anopheles mosquitoes [1, 2]. Malaria caused by* P. falciparum *is the deadliest form and the most predominant in Africa. The* P. vivax *is less dangerous but more widespread [3]. Symptoms of malaria are generally nonspecific and commonly consist of fever, malaise, weakness, gastrointestinal complaints (nausea, vomiting, and diarrhoea), neurological complaints (dizziness, confusion, and coma), and myalgia [4]. Others include headache, chills and/or cough, back and joint pains, delirium, convulsions, and anaemia [5].

Malaria continues to be a major public health concern in many countries of the world despite the efforts and progress made in reducing malaria cases and deaths. An estimated number of 214 million cases of malaria were recorded across the world in 2015 and this resulted in 438,000 malaria deaths, 90% of which occurred in Africa [6]. The disease burden is highest in poor, rural areas and deprived populations, contributing annually to an estimated 1.3% reduction in economic growth in high-burden countries [7], and reduces school attendance, impairs cognitive development in children, and lowers productivity [8].

Malaria can be effectively prevented and treated using tools that exist today [9]. A multipronged approach using insecticide treated nets (ITNs), indoor residual spraying (IRS), intermittent preventive treatment (IPT) of pregnant women and children, and prompt diagnosis and treatment using ACTs and other antimalarial agents such as quinine has been proven to prevent death and significantly reduce illness [10]. The way forward then is to improve education and sensitization of the populace, establishment of effective collaborations and the provision of the right tools, and improving access. Being largely a disease of poverty, malaria and its effects would be greatly reduced by improvement in the living conditions of the people. Others will include the adaptation of new approaches to the handling and treatment of malaria. The engagement and use of community health officers from both healthcare and social services sectors could help develop malaria plans and policies capable of providing preventive, promotional, and medical care to individuals and communities.

With children being the most vulnerable group among malaria patients, this study sought to identify the challenges and knowledge gabs associated with these interventions that would need to be addressed in order to improve and enhance malaria treatment outcomes, especially with the use of quinine in children.

## 2. Materials and Methods

### 2.1. Study Design

This was a cross-sectional study. The primary study method was the use of a well- structured questionnaire. It was carefully designed such that respondents had options to choose responses ranging from strongly agree, agree, neutral, and disagree to strongly disagree.

### 2.2. Study Site

This study was undertaken at a government children's hospital chosen because of its role in the healthcare delivery in the country. The hospital is located within the Ashiedu Keteke Sub- Metro area of the Greater Accra region. It is situated very close to the central business district of Ghana's capital, Accra, and very close to the popular Makola market, which facilitates easy access.

### 2.3. Study Population

Participants in the study were healthcare personnel, parents, guardians, and caregivers. These were selected as the target population because of their relationship with the child and their roles in paediatric healthcare delivery. A total of 748 participants were involved.

### 2.4. Sampling

Participants were purposively selected and included both men and women who were at least twenty years old at the time of sampling.

### 2.5. Ethical Considerations

Ethical approval for the study was obtained from the Committee on Human Research, Publication and Ethics, Kwame Nkrumah University Science and Technology, and Komfo Anokye Teaching Hospital (Ref: CHRPE/AP/017/17). Participants in the study were provided with the participant information leaflet and signed the consent forms prior to their participation. Those who could not read had it explained to them in their local dialect and their consent was thereafter given before they participated. The rationale of the study and all the issues concerning human ethics were carefully explained in the introduction to the online forms.

### 2.6. Data Collection Procedure

Data collection was made by a well-structured and self-administered questionnaire to obtain quantitative primary data. The study was conducted by the use of both hard copy and online questionnaire. The online questionnaire for quantitative data collection was via Internet based Google forms for the distribution of questionnaires and data collection through participant emails and web link invitations. Reminder emails were sent to the respondents during the data collection period to enhance respondent participation. In the case of participants with poor Internet connectivity and power cuts, respondents were given hard copies of the questionnaire with explanation of information contained in the leaflet to obtain due consent and followed up promptly for the collection of the filled-in questionnaire. Prior to the administration of the survey questionnaires, the duration, competency, and suitability of the questionnaires were pretested and returned a Cronbach's alpha reliability statistic of 0.904, an indication that the questionnaires were competent [11, 12].

The entire questionnaire was composed of two major parts. Part I took the biodata of the respondents. Part II on the other hand was subsectioned into various categories and captured data on the respondents' knowledge on malaria, preventive measures and treatment seeking attitude, paediatric drug formulations, antimalarial drug use options, quinine antimalarial therapy, and novel quinine formulations for children. The questionnaire (Part II) was designed in the form of Likert rating scale, from (1) strongly agree, (2) agree, (3) neutral, and (4) disagree to (5) strongly disagree. Participants indicated their level of agreement with the statements in each section by ticking the appropriate option.

### 2.7. Statistical Processing of Data

The SPSS version 20 was used to test for the internal consistency of the questionnaires to solicit the intended responses from the participants in the pretest. Data from 20 respondents obtained using Google forms (spreadsheets) were entered into the SPSS, and the Cronbach's alpha reliability statistic was determined.

The online forms were filled and returned, while responses from the hard copy questionnaires were filled manually into the Google forms. For the continuous data processing of the items in Part I, percentages were generated from the responses in the summary of the Google forms spreadsheet for the univariate analysis. Quantitative data for Part II was processed and automatically formatted into aggregated data charts for univariate analysis.

## 3. Results and Discussion

### 3.1. Biodata Distribution of the Study Participants

A total of 748 successfully completed questionnaires were obtained and comprised of 59% female and 41% male respondents. Majority of the respondents (53%) had attained tertiary education and 35% had secondary education, with the other 12% having left school at the primary level or other forms of basic education. Majority of the respondents (73%) were parents, guardians, or caregivers, while the other 27% were clinicians, nurses, and pharmacists. About 21% of the respondents were unemployed, but a greater proportion (79%) were either employed or self-employed (Table 1).

### 3.2. Causes and Modes of Malaria Transmission

**Figure 1:**
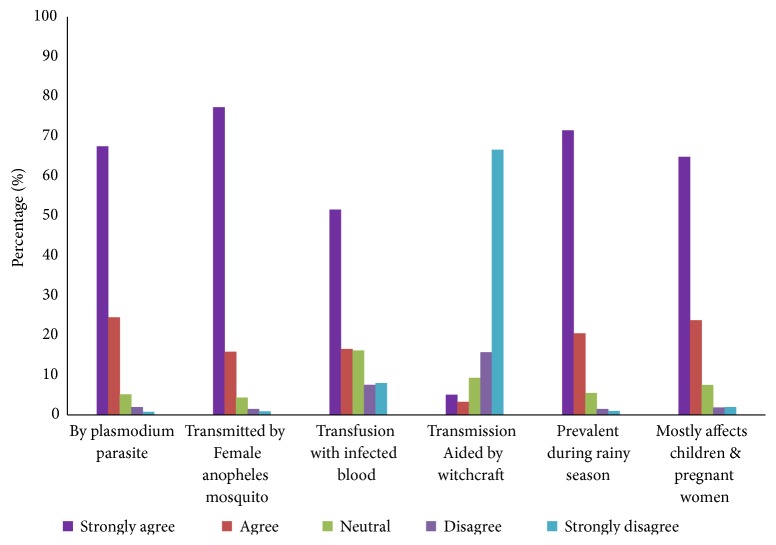
Awareness of the causes of malaria.

The study revealed that most of the respondents had a high level of understanding of the causes of malaria and its mode of transmission. Majority of the respondents associated malaria with the* Plasmodium *parasite (68%) and its spread through the bites of the infected female Anopheles mosquitoes (75%) (Figure 1). The extent of knowledge of the true causes of malaria from this study is similar to some earlier findings in Nigeria [13], Zimbabwe [14], Cameroon [15], and India [16], which all indicated an improvement over some related studies previously undertaken in Ghana and elsewhere. In those earlier findings, malaria was largely associated with some misconceptions such as drinking of dirty water, eating of oily foods, standing in the sun among others [17, 18].

Generally, the participants agreed that malaria cases become more widespread during the rainy season (75%) and identified children and pregnant women as the most vulnerable population (65%) for malarial attacks. Fortunately, the respondents (82%) indicated that malaria is not an evil disease as they dispelled the influence of witchcraft as a cause of malaria. The high knowledge level observed can be attributed to the educational levels of the participants in this study as some other findings also related education to one's awareness of malaria [19, 20].

Essentially, knowledge of the real causes and modes of transmission of malaria would be beneficial in the quest for malaria prevention.

### 3.3. Awareness of the Signs and Symptoms of Malaria

**Figure 2:**
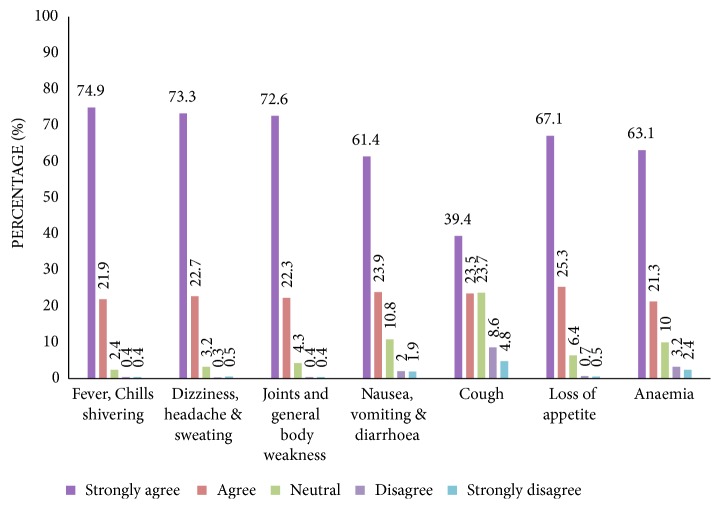
Knowledge of the signs and symptoms of malaria.

Majority of the respondents rightfully identified the signs and symptoms of malaria to include fever (75%), headache and dizziness (74%), general weakness (73%), nausea and vomiting (62%), loss of appetite (68%), and the resultant anaemia (63%) (Figure 2). These findings are similar to reports from some related works undertaken earlier on the subject [21, 22]. Knowledge of the right signs and symptoms would principally influence an individual's ability to take prompt and proper action at the onset of malaria, thereby triggering an appreciable treatment seeking attitude. The WHO and UK malaria treatment guidelines recommend early diagnosis and treatment as very essential in fighting malaria [23, 24].

Substantial understanding of the signs and symptoms of malaria can be crucial for home management of malaria and, hence, reduce severity and mortality. Therefore, even though there was considerable level of awareness of the clinical signs and symptoms of malaria, health authorities would need to intensify educational programmes among the populace aimed at maximum or near maximum malaria management and control.

### 3.4. Knowledge of the Methods of Malaria Prevention

**Figure 3:**
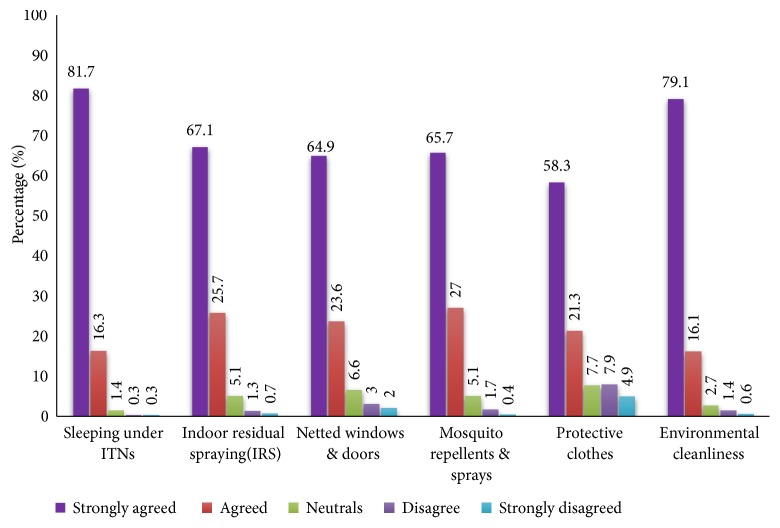
Awareness of the methods of prevention of malaria.

Malaria is deadly, yet it is an entirely preventable and treatable disease [2]. Methodologies range from the interference of breeding grounds and larva control to putting into practice of personal and household protective measures [25]. Generally, the knowledge about malaria prevention methods was high among the participants (Figure 3). While 97% agreed on the use of ITNs, 92% indicated IRS as means of preventing malaria. ITNs and IRS as well as environmental cleanliness have been used as malaria control mechanisms over the years [15].

Our study has also identified the use of mosquito repellents and netted windows and doors, while the wearing of protective clothes against mosquito bites was also emphasised, similar to several other study findings [26, 27]. Refreshingly, these measures are relatively cheaper than the use of IRS and ITNs [28] and can be implemented at ordinary household levels. The results established clearly that there is a relationship between the knowledge of the causes of malaria and its prevention. It also seems clear that the education and interventions by the National Malaria Control Programme is yielding the desired impact, and if intensified, the anticipated malaria cases will subside [27]. Therefore, children under 5 years and pregnant women in malaria endemic communities should continue to be targets in the malaria control programmes such as free or subsidized ITNs distribution and effective treatment via maternal and child health clinics.

### 3.5. Malaria Disease Management

The incidence of malaria was high in the study population, especially childhood malaria as 84% of respondents had children who had experienced malaria in the past. Further, 93% agreed that malaria is a serious and life-threatening disease which can kill if not treated promptly. The high level of knowledge of the deadly nature of malaria is encouraging, because proper measures would be taken to prevent or manage it should their family members get infected. Unfortunately, however, despite the fact that majority of the respondents had high level of knowledge on apt malaria prevention methods, their children (84%) still had episodes of malaria infection at one point or the other.

It therefore appears that the challenges to malaria prevention are still enormous and complicated. Lack of adequate protective clothing, the hot and humid Tropical African weather which does not support or encourage thorough body covering and use of ITNs, abundance of the mosquito vector in the subregion coupled with changes in their feeding times and habits, lack of effective IRSs, or the mere inability to implement the knowledge on malaria prevention exacerbates these challenges. Mboera et al. [29], in a similar study observed that in spite of high levels of knowledge of malaria in Tanzania, its morbidity was still high, and they suggested that misguided application of antimalarial drugs and delayed health seeking attitude as well as reliance on clinical findings in the absence of laboratory confirmation were accountable.

Treatment seeking attitude was good although pockets of participants agreed on undertaking home treatment with either previously bought drugs or herbal products. Essentially, majority of the respondents sought formal healthcare services from hospitals, clinics, or the community health workers [30], although some other studies [31] contradict this in favour of home treatment of some kind.

Interestingly, the study showed that 77% of the participants would seek treatment within 24 hours once symptoms showed up, which is in line with the targets of the Abuja Summit on Malaria which among other things envisaged that at least 60% of those having malaria should seek treatment within 24 hours of the onset of symptoms [32].

Nevertheless, some of the participants identified long distances to health facilities, high transportation, consultation and treatment costs, unfriendly attitude of some healthcare workers, unnecessary delays at the health facilities, and busy work schedules as some of the reasons for not seeking formal healthcare (Table 2).

Some form of continuing health education is still necessary to help these parents understand the need to take their children to healthcare centres for treatment in spite of these difficulties and challenges. Maybe the introduction of some basic incentives for prompt healthcare seeking attitudes, especially with children and pregnant women, could greatly enhance this. Also, since malaria is still a major killer in Africa, there is the need to restructure the health systems to facilitate the use of community health officers (CHOs) to take malaria control to the populace rather than wait and expect them to use the health facilities, in view of the high level of poverty and low literacy in these communities. The use of CHO services has been widely demonstrated to be very effective and have resulted in the elimination of most childhood killer diseases in many communities, improving family planning services and lifestyles of, especially, mothers and children [33].

Meanwhile some health workers in the various centres would need a rethink in attitude towards patients and guardians who seek healthcare services from them. This may require further training and continuous professional development, where some of these findings are conveyed to these practitioners.

### 3.6. Desirable Attributes of Paediatric Drug Formulations

The use of off-label and unlicensed medicines in children is widespread, yet the effects of these medicines on children have not been properly studied. Healthcare professionals, parents, or caregivers are often confronted with the necessity to manipulate an adult medicine for children which has the potential for high levels of dosage errors, instability, poor bioavailability, and unintended side-effects. Children under five years have been the target for antimalarial drug therapies over the years. The availability and suitability of antimalarial drug formulations for the child are essential if the fight against paediatric malaria is to be won.

Suitable formulations for children make it easier for parents and caregivers to administer antimalarial agents effectively, resulting in better therapeutic outcomes [34, 35]. Paediatric formulation ought to satisfy the needs: convenient and reliable administration, acceptability and palatability, minimum dosing frequency, end-user needs of patients of diverse ages, and the preferences of patients including caregivers in different parts of the world [36, 37].

**Figure 4:**
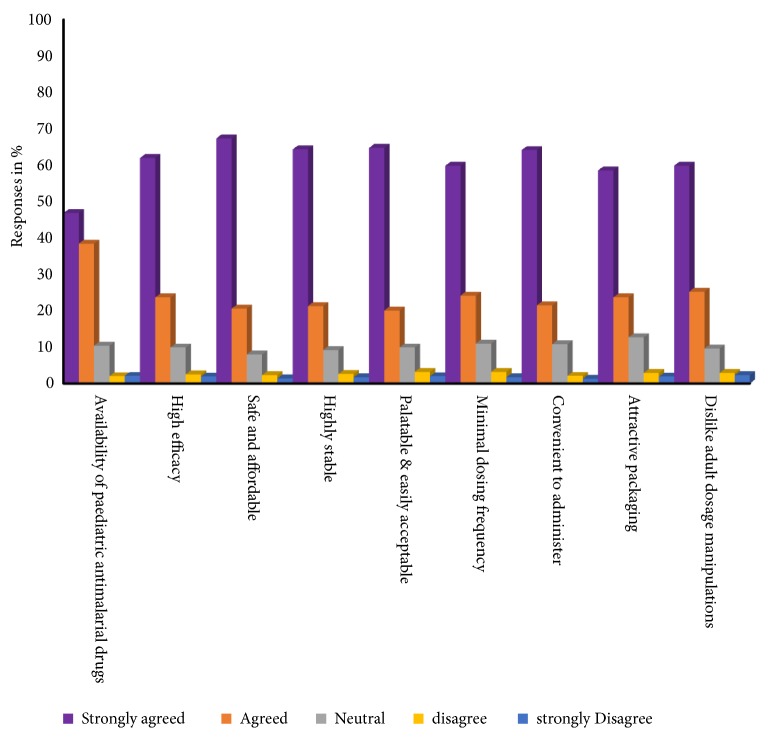
Desirable attributes of paediatric drug formulations.

Age-appropriate dosage forms are necessary to ensure compliance and effective therapeutic outcomes [38]. Liquid formulations hitherto had been the formulation of choice for paediatric use but issues of compliance and safety have been expressed. Liquid formulations can easily undergo microbial contamination if proper storage conditions are not followed. Most antimalarial agents also have unpleasant taste and bitter tasting drugs are usually poorly tolerated by children. A significant proportion (85%) of the study participants were aware of some paediatric antimalarial drug formulations in circulation and showed preference for formulations which are palatable, easily acceptable, and in an attractive packaging (Figure 4).

Palatability and dysphagia persist as the two paramount challenges to paediatric drug product development, because neither is trivial and therefore continues to be major obstacles to the development of ‘child-friendly' dosage forms [39].

### 3.7. Antimalarial Drug Knowledge

The study uncovered that some antimalarial drugs were more common among the participants than others (Table 3). Quinine came topmost (92%) followed by chloroquine (90%) as the antimalarial agents most of the respondents were familiar with. This may be because of the unique bitter taste of the duo as many people are more likely to remember bad taste. The availability of quinine tonic on the market may have an influence. In addition, quinine has been in use for treating malaria for over two centuries now and so the awareness of it among the respondents is fairly understandable. Unfortunately, chloroquine, though well known, has fallen into disuse and is no longer recommended due to drug resistance resulting from dosing errors, inappropriate use, or misuse and abuse.

The knowledge of the respondents on the ACTs was appreciable, but artesunate-amodiaquine (92%) and artemether-lumefantrine (87%) were the most common among the respondents (Table 3). Lonart (92%), Coartem (87%), Lumether (78%), and Malar-2 (67%) were, respectively, the leading marketed brands of ACTs, in respect of the knowledge of the respondents. The Governments' recent policy on the use of ACTs and the education and advertising that have gone on with it could have played a major role in this outcome. The challenges here are the lack of proper understanding of the term ACT and the proliferation of numerous brands with varying efficacy and effectiveness. In addition, treatment failures with these unreliable brands have emphasised the use of quinine.

### 3.8. Knowledge of Quinine Antimalarial Therapy

Quinine has been an age-old drug used for the treatment of malaria. Although, the WHO does not recommend it as a first line antimalarial drug for uncomplicated malaria, quinine is still used in the treatment of severe malaria and in cases where the ACTs fail to give the required therapeutic outcome. It was known among majority of the study participants that quinine is used for treating both complicated and uncomplicated malaria (Table 4), as supported by the UK malaria treatment guidelines [23].

Quinine, a cinchona alkaloid, has been an important antimalarial agent for a long time and continues to be effective against chloroquine-resistant* P. falciparum *malaria [40]. In settings where multidrug resistant strains prevail, a 7-day regimen of quinine plus tetracycline or doxycycline or clindamycin gives cure rates of over 90% for patients with* P. falciparum *malaria [41]. Quinine use in children is chiefly in the form of syrups which are sweetened because of its bitter taste. There are no other paediatric formulations of quinine, and when treating children, care should be taken to ensure correct doses are administered and retained because of its bitter taste and the fear of vomiting.

The unpalatability of quinine syrups as well as the crushed tablets may lead to the use of subtherapeutic dosages, noncompliance, and potential development of parasitic resistance. The study has revealed that the participants were familiar with the difficulties regarding quinine use in children, such as the bitter taste, and agreed that the bitterness of quinine is affecting their children's acceptance of the drug. As such the oral administration of large of volumes of quinine syrup may be difficult to achieve in children. Hence, 89% of the respondents agreed that they would be happy if the bitterness of quinine can be masked. This poses a challenge and places a burden of trust and responsibility on academia and industry players to act decisively and quickly. The WHO initiative “Make Medicine Child Size”, aimed at increasing awareness and access to child specific medicines [42], and the European Paediatric Formulation initiative [43] both seek to promote the preparation of effective and safe children medications by facilitating the sharing of expertise between key stakeholders in the health industry. These are aimed at identifying and dealing with common challenges in developing formulations for paediatric populations. Ideally, formulations meant for children should possess minimal frequency of dosing, insignificant impact on lifestyle, minimum safe excipients, and wide range of applicability and should as well be easy to produce, stable, elegant, cost-effective, and commercially available [44, 45].

### 3.9. Choice of Novel Oral Paediatric Quinine Formulations

**Figure 5:**
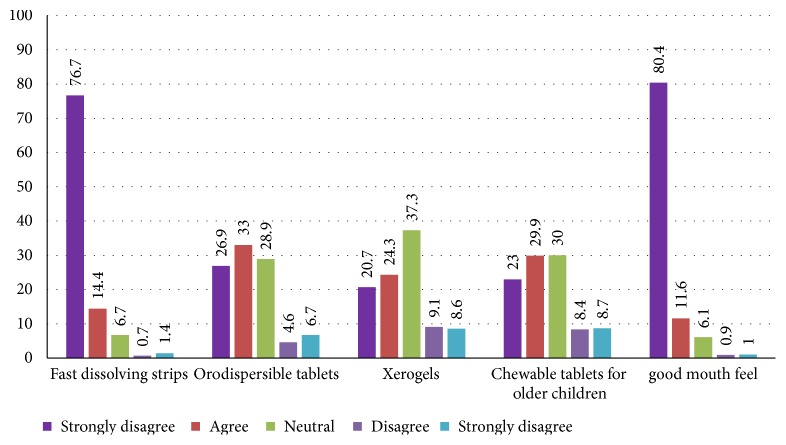
Novel paediatric quinine formulations.

As a solution to some of the challenges enumerated with respect to quinine use in children, the study sought to determine the formulation of choice of quinine for paediatric use. It became clear that majority of the participants (91%) generally favoured oral thin film/strip formulation and some 92% of the respondents agreed that they would appreciate a formulation that guarantees a good mouth feel (Figure 5).

Fast dissolving oral thin films have become the cutting-edge form of oral solid dosage forms in view of more flexibility and comfort [46]. They are more easily tolerated by patients and caregivers with respect to their ease-of-delivery, accurate dosing, and portability [47–49]. It enhances the dissolution and efficacy of drugs in a matter of seconds in the oral cavity once it gets into contact with saliva and therefore requires no water or mastication for administration [50, 51].

The predetermined form of oral dissolving thin films (ODTFs) offers an accurate and easily swallowed dose without water that allows for portable and convenient “give and go” administration by a caregiver or parent [52]. It facilitates quick absorption and instant bioavailability of drugs due to high blood flow and permeability within the oral cavity.

Fast dissolving thin films are particularly useful in patients of such groups as paediatric, geriatric, emetic, and the bedridden [53, 54]. Oral thin films have the fundamental benefit of accuracy of dosing as compared to liquids, and there is no fear of choking as in the case of tablets and capsules. The novelty of the oral thin film is that it dissolves rapidly in the mouth invariably within 10–15 seconds [55].

Therefore, quinine in ODTFs with a good ‘mouth feel' will provide the key to an efficient and effective use of quinine in childhood malaria.

## 4. Conclusion

The study demonstrated the availability of substantial knowledge of various aspects of malaria as well as its devastating burden, especially in children. Yet, the significant knowledge did not yield much in reduction of malaria episodes. The study has thus emphasised the need to ensure that tools such as insecticides treated nets and efficacious antimalarial agents are made available and utilised effectively in the fight against malaria. The availability of appropriate formulations of paediatric antimalarial agents such as quinine and its right usage would be key in any future malaria control strategies. The formulation of choice for quinine in the light of this study is the fast dissolving oral thin film which comes along with it efficient taste masking opportunities as well as guaranteeing a good mouth feel. Additionally, to control malaria some level of education is needed. Populations that demonstrate incorrect knowledge of malaria and its therapy may be illiterate or possess a low level of formal education. Therefore, there is a need for continued education since malaria control has so many facets and education is key to its success. Malaria control should be handled in a systematic, multisectoral, and coordinated manner to be effective.

## Figures and Tables

**Table 1:** Sociodemographic distribution of the study participants.

**Characteristic**	Frequency (n)	Percentage (%)
**Age range in years**		
20 – 30	267	35.69
31 – 40	256	34.23
41 – 50	123	18.05
51 – 60	64	9.22
61 – 70	17	2.27
70 +	3	0.54

**Gender**		
Male	303	40.51
Female	455	59.49

**Marital status**		
Single	289	38.64
Married	459	61.36

**Education level**		
Primary	74	9.89
Secondary	265	35.43
Tertiary	393	52.54
Other	16	2.14

**Employment status**		
Unemployed	159	21.26
Self-employed	279	37.30
Employed	310	41.44

**Role in care giving**		
Healthcare professional	199	26.60
Parent/guardian/caregiver	549	73.40

**Table 2:** Malaria treatment interventions and healthcare seeking attitude.

Treatment interventions & healthcare seeking attitude	Strongly agree (%)	Agree (%)	Neutral (%)	Disagree (%)	Strongly disagree (%)
Malaria affected my child in the past	54.4	29.3	8.7	3.1	4.5
Malaria is deadly if not treated	79.4	14	3.9	1.3	1.3
***Treatment seeking attitude***					
I do home treatment	24.5	23.4	26.9	13.6	11.6
Use herbal drugs in home treatment	17.9	21	29.1	14.8	17.1
Drugs bought previously	17.5	24.2	26.2	15.9	16.2
Begin treatment within 24 hrs	55.6	21.1	13.6	5.2	4.4
Begin treatment after three days	6	7.8	22.1	26.7	37.4
Child will get better after sometime so I just wait	4.4	6.6	21.8	19.3	48
***Outcome of home treatment***					
Home treatment successful	15.8	21.7	44.5	9.8	8.3
Home treatment made illness worse	8	21.4	49.2	14	7.4
Home treatment failed	16.6	17.9	44.3	11.6	9.6
***Reasons for seeking healthcare***					
I expect blood test to be done at the hospital	53.5	31.7	8.7	3.5	2.7
I expect prompt action at the health facility	67.6	20.6	6.4	3.2	2.1
***Source(s) of healthcare outside the home***					
I seek healthcare at hospital/clinic	56.1	25.8	11.1	3.1	3.9
Healthcare from community health worker	16.8	31	25.8	12	14.3
Traditional/spiritual leaders for divination & treatment	2.1	4.8	11.6	18.7	62.7
Religious leaders for prayers	6.3	4.5	12.3	16.7	60.2
Hospital if home treatment fails	13.4	19.9	23.4	17	26.3
***Reasons for not seeking healthcare***					
Unnecessary delays at hospitals	25.9	24.7	26.9	10.4	12
Unfriendly attitude of health some workers	24.1	25.3	27.7	11.9	11.1
Long distances to health centres	13	17.5	37.6	18	13.9
High cost of transportation, consultation and treatment	26.3	23.7	21.5	14.2	14.3
Busy work schedules	10.4	13.2	34.1	22.6	19.7
I buy drugs from pharmacy	20.5	30.6	27.3	9.9	11.8

**Table 3:** Knowledge on the availability of some antimalarial drugs.

**Antimalarial drug**	**Strongly agree (**%**)**	**Agree (**%**)**	**Neutral (**%**)**	**Disagree (**%**)**	**Strongly disagree (**%**)**
Chloroquine	57.5	32.8	5.5	1.5	2.8
Quinine	66	25.8	5.2	1.3	1.6
Amodiaquine	59.6	30.5	6.3	1.7	1.9
Halofantrine	17	16.7	41.6	13.8	11
Primaquine	12.7	16.3	44.1	14.6	12.3
Mefloquine	11.6	17.6	43.9	2.7	12.4
Artesunate	57.1	22.5	12.6	3.1	4.8
Dihydroartemisinin	18.9	23.8	34.4	13.2	9.8
Piperaquine	12.4	24.6	36.8	14.3	11.9
Sulfadoxine & Pyrimethamine	19.5	20.9	34.6	12.7	12.3

*ACTs*					

Artesunate + amodiaquine	69.5	22.5	4.3	1.3	2.4
Artemether + lumefantrine	61	25.5	8.2	2.4	2.9
Dihydroartemisinin + piperaquine	21.1	25.3	33.2	11.9	8.6
Artesunate + Mefloquine	10.8	21.4	40.1	16.8	10.8
Artesunate + Sulfadoxine & Pyrimethamine	12.2	22.2	39.3	15.6	10.8

*Marketed Brands of ACTs*					

Lonart	63.8	28.5	4.5	1.1	2.1
Coartem	57.8	29.4	7	2.5	3.3
Lumether	50.9	27.5	14.3	3.3	3.9
Malar-	43.3	23.7	19.7	7	6.4
P-Alaxin	35.3	24.9	24.5	8	7.4
Danmether	31	20.5	31	9	8.6
Gen-M	34.5	15.6	25.4	9.8	14.7

**Table 4:** Knowledge on quinine antimalarial therapy.

Knowledge on quinine antimalarial therapy	Strongly agree (%)	Agree (%)	Neutral (%)	Disagree (%)	Strongly disagree (%)
I've given quinine to a child for malaria before	38.4	27.3	16	7.4	11

Quinine treats both complicated and uncomplicated malaria	35.4	41.4	14.2	3.3	5.6

Quinine has a bitter taste	70.5	17.9	7.4	1.6	2.7

Quinine's bitterness is affecting my child's acceptance	59.1	25.7	10.6	1.2	3.5

I will be happy if the bitterness can be masked	71.9	16.6	7.1	1.6	2.8

Tinnitus, headache, nausea, etc. appear after taking quinine	23.3	26.2	37	4.9	8.6

Oral administration of large volumes of quinine syrup may be difficult to achieve	58.8	24.7	10.3	2.5	3.6

## Data Availability

The data used to support the findings of this study are available from the corresponding author upon request.

## Abbreviations

ACTs: Artemisinin-based combination therapies
IRS: Indoor residual spraying
IPT: Intermittent preventive treatment
ODTFs: Oral dissolving thin films
WHO: World Health Organization.
